# Neurodegeneration, Neurogenesis, and Oxidative Stress

**DOI:** 10.1155/2013/730581

**Published:** 2013-11-24

**Authors:** Renata Santos, Carmen Ruiz de Almodóvar, Anne-Laure Bulteau, Cláudio M. Gomes

**Affiliations:** ^1^Development of the Nervous System, IBENS, Ecole Normale Supérieure, 46 rue d'Ulm, 75230 Paris Cedex 05, France; ^2^Biochemistry Center, University of Heidelberg, Im Neuenheimer Feld 328, 69120 Heidelberg, Germany; ^3^IPREM, University of Pau, 2 rue du President Angot, 64000 Pau, France; ^4^Instituto Tecnologia Quimica e Biologica, Universidade Nova de Lisboa, Avenida da República, EAN, 2784-505 Oeiras, Portugal

Oxidative stress is implicated in the pathophysiology of a wide variety of neurodegenerative and neurologic disorders. This special issue includes 11 articles that cover different aspects of the importance of oxidative stress in neurotoxicity, neurodegeneration, and potential therapies.

Neurotoxicity can occur after exposure to natural or artificial molecules that alter the normal function of the nervous system. The review article by R. Lugo-Huitrón et al. focuses on the toxicity of quinolinic acid, an endogenous metabolite of the kynurenine pathway, normally present in the brain and cerebrospinal fluid. This paper discusses the different mechanisms by which quinolinic acid acts as a neurotoxin, such as the activation of N-methyl-D-aspartate (NMDA) receptors, mitochondrial dysfunction, oxidative stress, inflammation, and cell death. B. Wang and Y. Du review the *in vitro* and *in vivo* evidence of the neurotoxic effects of cadmium, a heavy metal, in the central nervous system.

Several original and review articles examine the role of oxidative stress in diverse neurodegenerative diseases. The original article by C. Savino et al. shows the implication of p66Shc protein and the mitochondrial permeability transition pore pathway in neurodegeneration in a mouse model of autoimmune encephalomyelitis. The role of mitochondria in neurodegeneration is further discussed in two review articles by V. García-Escudero et  al. and by Y. Zhao and B. Zhao. These authors present data on the role of mitochondrial dysfunction and oxidative stress in the initiation and progression of Alzheimer's disease, respectively. H. Ariga et al. describe the function of DJ-1 in the pathogenesis of Parkinson's disease. Furthermore, P. Milani et al. summarize the recent literature describing the interaction between Nrf2, a master regulator of the antioxidant response, and DJ-1 and SOD1 proteins and its interest in the development of therapeutic strategies for Parkinson's disease and amyotrophic lateral sclerosis. Two review articles focus on Friedreich's ataxia disease. C. M. Gomes and R. Santos overview the data supporting that oxidative stress is a central feature of this disease. In an opinion paper A. Bayot and P. Rustin present arguments in favor of the hypothesis that the reduction in the expression of *PIP5K1B*, the gene adjacent to the frataxin-encoding gene, in Friedreich's ataxia patient cells is implicated in disease onset and progression.

The antioxidant effect of numerous phytochemicals has long been recognized and has been proposed as an alternative form of treatment, since still no drugs are available that prevent the progression of most neurodegenerative diseases. In an original article, H. Mo et al. describe the potential neuroprotective effect of tea polyphenols in an *in vivo* subarachnoid hemorrhage mice model. A. Tarozzi et al. present a review of the antioxidant properties of sulforaphane primarily attributed to its ability to activate the Nrf2/ARE pathway.

We believe that these contributions provide an updated view on this dynamic field and evidence that supports the importance of considering oxidative stress as a player in neurodegenerative diseases as well as neurotoxicity. 

